# *Msx1 *and *Msx2 *are required for endothelial-mesenchymal transformation of the atrioventricular cushions and patterning of the atrioventricular myocardium

**DOI:** 10.1186/1471-213X-8-75

**Published:** 2008-07-30

**Authors:** Yi-Hui Chen, Mamoru Ishii, Henry M Sucov, Robert E Maxson

**Affiliations:** 1 Department of Biochemistry and Molecular Biology, USC/Norris Comprehensive Cancer Center and Hospital, Keck School of Medicine, University of Southern California, 1441 Eastlake Avenue, University of Southern California, Los Angeles, CA 90089-9176, USA; 2 Institute for Genetic Medicine, Keck School of Medicine, University of Southern California, 2250 Alcazar Street, Suite 240, Los Angeles, CA 90089-9075, USA; 3 Stem Cell Laboratories, Genomics Research Center, Acadmia Sinica, Taipei 11529, Taiwan

## Abstract

**Background:**

*Msx1 *and *Msx2*, which belong to the highly conserved *Nk *family of homeobox genes, display overlapping expression patterns and redundant functions in multiple tissues and organs during vertebrate development. *Msx1 *and *Msx2 *have well-documented roles in mediating epithelial-mesenchymal interactions during organogenesis. Given that both *Msx1 *and *Msx2 *are crucial downstream effectors of Bmp signaling, we investigated whether *Msx1 *and *Msx2 *are required for the Bmp-induced endothelial-mesenchymal transformation (EMT) during atrioventricular (AV) valve formation.

**Results:**

While both *Msx1-/- *and *Msx2-/- *single homozygous mutant mice exhibited normal valve formation, we observed hypoplastic AV cushions and malformed AV valves in *Msx1-/-; Msx2-/- *mutants, indicating redundant functions of *Msx1 *and *Msx2 *during AV valve morphogenesis. In *Msx1/2 *null mutant AV cushions, we found decreased Bmp2/4 and *Notch1 *signaling as well as reduced expression of *Has2*, *NFATc1 *and *Notch1*, demonstrating impaired endocardial activation and EMT. Moreover, perturbed expression of chamber-specific genes *Anf*, *Tbx2*, *Hand1 *and *Hand2 *reveals mispatterning of the *Msx1/2 *double mutant myocardium and suggests functions of *Msx1 *and *Msx2 *in regulating myocardial signals required for remodelling AV valves and maintaining an undifferentiated state of the AV myocardium.

**Conclusion:**

Our findings demonstrate redundant roles of *Msx1 *and *Msx2 *in regulating signals required for development of the AV myocardium and formation of the AV valves.

## Background

A complex series of morphogenetic events and hemodynamic influences are required for cardiogenesis [1-3]. Malformations of cardiac valves constitute the most prevalent form of human birth defects, appearing in nearly one percent of newborn infants [4,5]. The formation of cardiac valves requires two major consecutive steps: cardiac cushion formation and valve remodeling [1,4,6]. After cardiac looping, the cardiac cushions in the regions of the atrioventricular (AV) canal and distal outflow tract (OFT) are formed through an endothelial-mesenchymal transformation (EMT), a remarkably complex event initiated by the specification and activation of a subset of endothelial cells in the cushion-forming regions. This event is followed by cell delamination from the endocardium and cell migration into the extracellular matrix between the endocardium and myocardium (referred to as the cardiac jelly) [4-6]. Concomitant with migration into the cardiac jelly, the endothelial cells transdifferentiate into mesenchymal cells and proliferate to form multiple layers, resulting in the expansion of the cushion crests toward each other [6]. AV cushion formation is followed by a series of morphogenetic events, including elongation, outgrowth and remodeling, which result in the conversion of the thick cushions into thin valve leaflets [1,6-8]. Several signaling molecules have been implicated in regulating EMT during cardiac valve formation, including the Nuclear Factor in Activated T cells (NFAT) [9-12], Vascular Endothelial Growth Factor (VEGF) [11,13], and members of the Epidermal Growth Factor (EGF) [1,6,14,15], Bone Morphogenetic Protein (Bmp) [16-19], Notch [20-22], Transforming Growth Factor-β (TGF-β) [4,18,19,23], and Wnt/β-catenin families [24,25].

*Msx1 *and *Msx2*, closely related members of the *Nk*-family of homeobox transcription factors, have well-documented roles as both downstream effectors and upstream regulators of Bmp signalling [26-29]. *Msx1 *and *Msx2 *function redundantly in multiple tissues and organs during vertebrate development, including the heart [30,31]. We showed previously that *Msx1-/-; Msx2-/- *mutants exhibit malalignment defects of the developing outflow tract including double outlet right ventricle and pulmonary atresia or stenosis [30,32]. These defects are associated with excessive proliferation of cardiac neural crest, endothelial and myocardial cells in the mutant outflow tract between E10 and E11 [32].

Reduced expression of *Msx1 *and *Msx2 *was observed in the AV cushions deficient in Bmp signaling. Such cushions also displayed immature cardiac jelly, compromised AV myocardium and hypoplastic AV valves [17,33]. However, no valve defects have been reported in mice deficient in either *Msx1 *or *Msx2 *[34,35].

In the present study, we focused on AV cushion and valve formation in mice with combined deficiencies of *Msx1 *and *Msx2*, and compared marker gene expression in *Msx1-/-; Msx2-/- *double mutant AV cushions with that in *Msx1-/- *and *Msx2-/- *single mutant AV cushions. We observed hypoplastic AV cushions and deformed AV valves in *Msx1-/-; Msx2-/- *double mutants but not in *Msx1-/- *or *Msx2-/- *single mutants, and no discernable difference in the expression of AV cushion markers between wild-type and single mutant mice. On the other hand, there was a reduced level of NFATc1 immunostaining in the *Msx1/2 *double mutant AV endocardium, and decreased expression of α-smooth muscle actin, *Notch1*, *Has2*, *Bmp2/4 *and *Pitx2 *in the *Msx1/2 *double mutant AV cushion mesenchyme, indicating impaired EMT and cushion formation [4,9,10,12,16,17,21,22,36-41]. In addition, perturbed expression of *Bmp2/4*, *Tbx2*, *Anf*, *Hand1 *and *Hand2 *in the *Msx1/2 *null mutant AV myocardium suggests impaired myocardial patterning during chamber formation [17,42-49]. Taken together, we conclude that *Msx1 *and *Msx2 *function redundantly in regulating the expression of genes required for AV canal (AVC) morphogenesis.

## Results

### Hypoplastic AV cushions and impaired endocardial signaling in Msx1-/-; Msx2-/- mutant hearts

**Figure 1:**
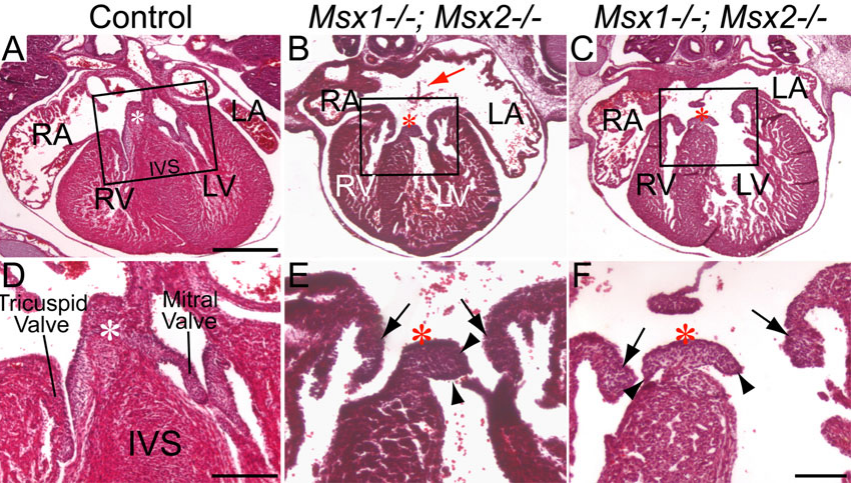
**Hypoplastic AV cushions and disorganized AV valves in *Msx1-/-*; *Msx2-/- *double mutants at E15.5**. Panels D-F are enlarged views of the boxes in panels A-C from histological sections of E15.5 embryonic hearts. Compared with the control (D), *Msx1/2 *null mutants have compromised AV cushions (indicated by white asterisks in the control and red asterisks in *Msx1/2 *double mutants), shorter and deformed AV valves extending from the cushions (indicated by triangle arrowheads in panels E and F), and hyperplastic AV valves extending from the AV myocardium (indicated by arrows in panels E and F), suggesting impaired valve outgrowth and remodeling following deficient EMT to form the AV cushions. The red arrow indicates atrial septal defect. IVS, interventricular septum; LA and LV, left atrium and ventricle; RA and RV, right atrium and ventricle. Scale bars: 0.5 mm in A (for A-C) and 0.2 mm in D, and F (for E, F).

Histological sections of hearts between E14.5 and E16.5 revealed atrial septal defect as well as deformed AV cushions and valves in *Msx1-/-; Msx2-/- *double mutants (*n *= 8), but not in *Msx1-/- *or *Msx2-/- *single mutants (*n *= 22), nor in *Msx1-Msx2 *homozygous-heterozygous compound mutants (*n *= 20). All eight *Msx1/2 *null mutants examined had hypoplastic AV cushions and shorter but thickened AV valves, indicating a failure to undergo proper valve outgrowth and remodeling (Fig. 1B, C, E and 1F).

**Figure 2:**
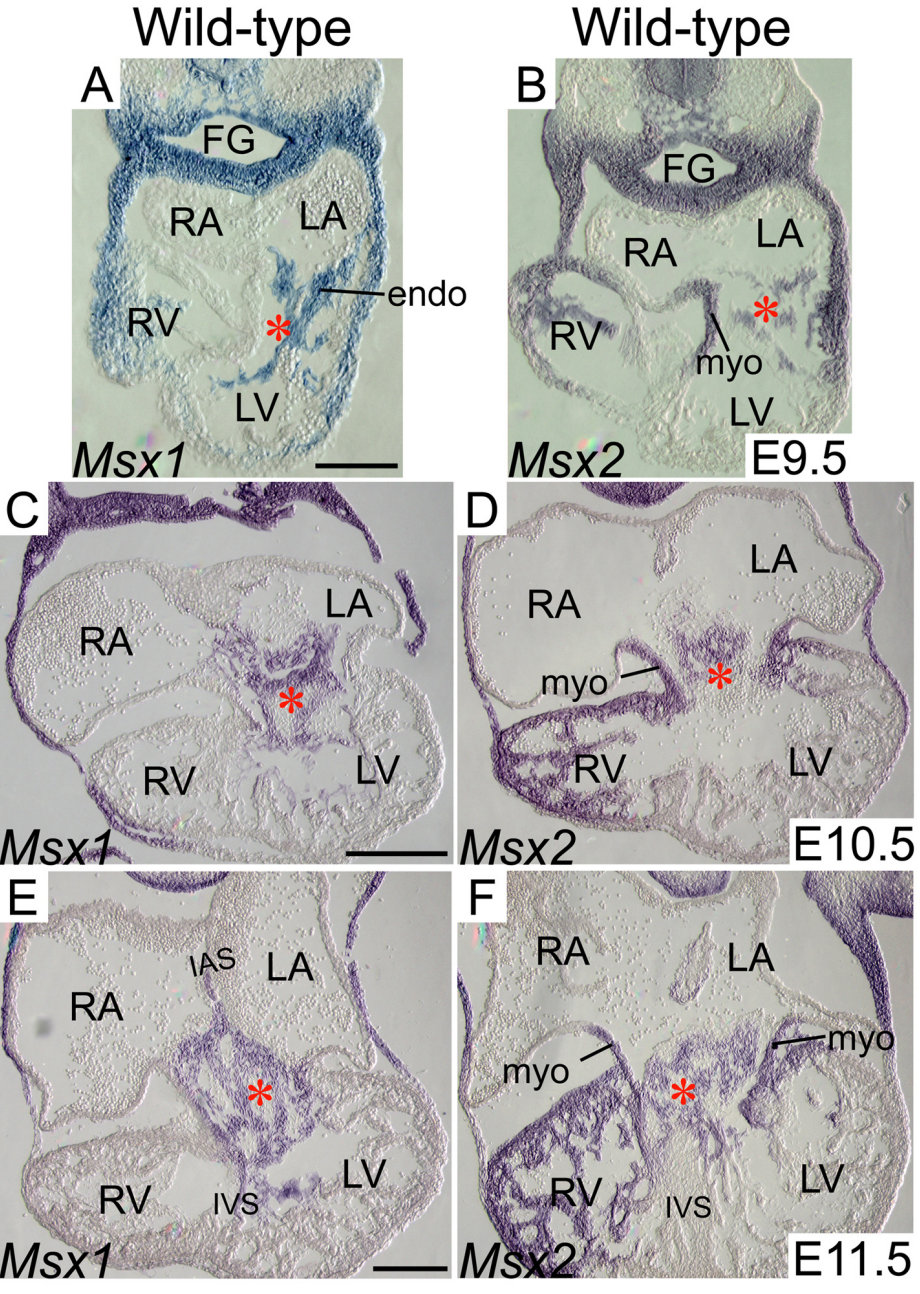
**Expression of *Msx1 *and *Msx2 *in the developing AV canal**. Section in situ hybridization showed that the domains of *Msx1 *and *Msx2 *expression overlap in a subpopulation of endocardial and cushion mesenchymal cells during AV cushion morphogenesis between E9.5 and E11.5 (A-F). *Msx2 *also displayed strong expression in the myocardium of the AV canal and right ventricle at all developmental stages examined (B, D and F). Red asterisks indicate AV cushions. endo, endocardium; FG, foregut; IAS and IVS, interatrial and interventricular septum; LA and LV, left atrium and ventricle; myo, myocardium; RA and RV, right atrium and ventricle. Scale bars: 0.1 mm in A (for A, B), 0.2 mm in C (for C, D) and E (for E, F).

To evaluate the functional redundancy of *Msx1 *and *Msx2 *in AV cushion morphogenesis, we examined expression patterns of *Msx1 *and *Msx2 *in the AV canal (AVC) between E9.5 and E11.5, while EMT of the endocardial cushions was actively taking place [4,16,18]. At E9.5 and E10.5, *Msx1 *and *Msx2 *displayed overlapping expression in the endocardial and cushion mesenchymal cells, while *Msx2 *was also expressed in the AV myocardium, where no *Msx1 *expression was detected (Fig. 2A–D). By E11.5, *Msx1 *expression extended to the lateral cushion mesenchyme adjacent to the AV myocardium as well as the membranous parts of the interatrial and interventricular septa (Fig. 2E). Our observations suggest that *Msx1 *and *Msx2 *may function redundantly during AV endocardial EMT and AV cushion morphogenesis.

**Figure 3:**
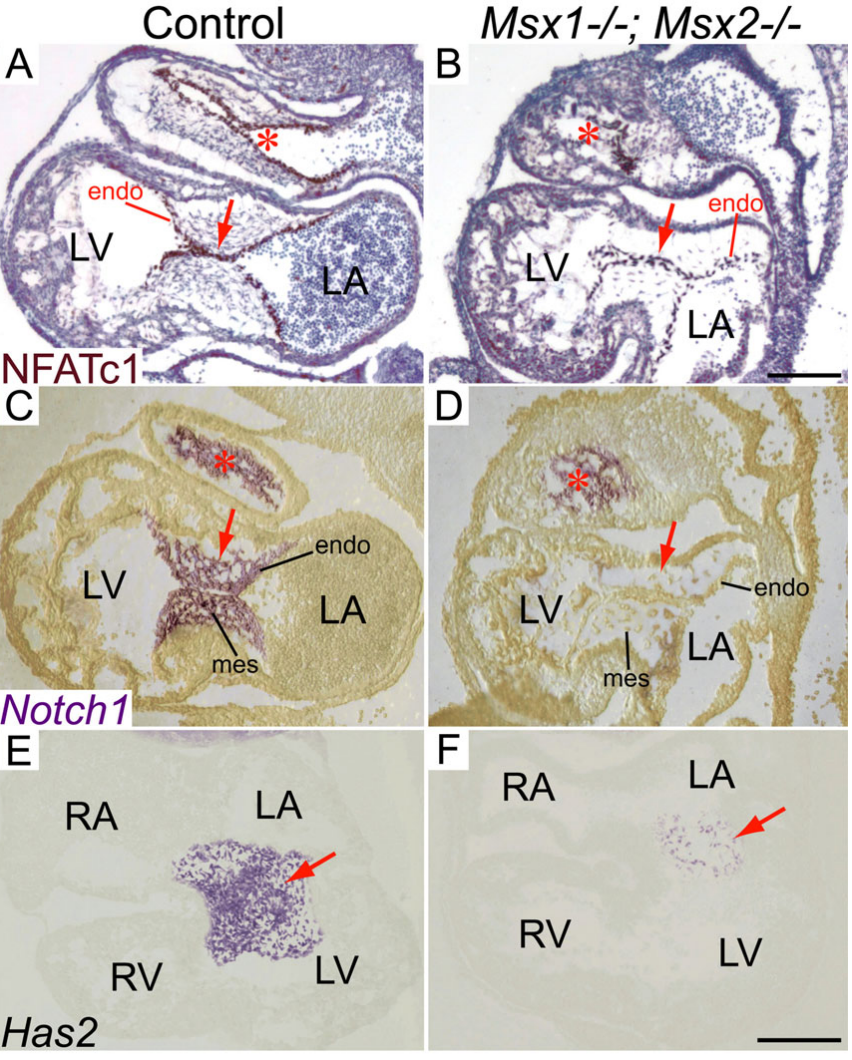
**Impaired expression of endocardial and cushion mesenchymal genes in *Msx1-/-*; *Msx2-/- *mutant AV cushions**. At E10.5, the immunostaining intensity of NFATc1 was reduced in *Msx1-/-*; *Msx2-/- *mutant AV endocardium compared with the littermate control (red arrows in A, B), while the intensity was comparable between control and *Msx1/2 *double mutant OFT endocardium (red asterisks in A, B). At the same developmental stage, RNA section in situ hybridization revealed greatly diminished expression of *Notch1 *(arrows in C, D) in the *Msx1/2 *double mutant AV cushion mesenchyme and endocardium compared with the control. In contrast, *Notch1 *expression was normal in the *Msx1/2 *mutant OFT cushions (compare red asterisks in C and D). Note also a decreased number of *Has2*-expressing cells in the *Msx1/2 *null mutant AV cushion mesenchyme (red arrows in E, F). endo, endocardium; LA and LV, left atrium and ventricle; mes, mesenchyme; RA and RV, right atrium and ventricle. Scale bars: 0.1 mm in B (for A-D), 0.2 mm in F (for E, F).

We then studied whether endocardial cell maturation and activation were perturbed in *Msx1/2 *double mutants. Immunostaining for NFATc1 (nuclear factor in activated T cells), which was previously shown to be upregulated in activated endothelial cells within the AVC [10,12,17], indicated that NFATc1 was present in the *Msx1/2 *double mutant AV endocardium at E10.5, but with a significantly lower staining intensity compared to the control AV endocardium (compare the regions pointed by red arrows in Fig. 3A and 3B). In contrast, the intensity of NFATc1 immunostaining in the OFT endocardium was comparable between controls and *Msx1-/-; Msx2-/- *mutants (compare the regions indicated by red asterisks in Fig. 3A and 3B). At the same developmental stage, the expression of *Notch1*, an essential inducer of EMT [4,21,22], was greatly diminished in the *Msx1/2 *double mutant AV cushions compared with the control (compare the regions pointed by red arrows in Fig. 3C and 3D). In contrast, *Notch1 *expression in the *Msx1/2 *double mutant OFT cushions was comparable with that in the control OFT (compare the regions marked by red asterisks in Fig. 3C and 3D). On the other hand, the expression of another endocardial marker gene *Twist1 *in *Msx1-/-; Msx2-/- *double mutants was comparable with that in control embryos (data not shown).

**Figure 4:**
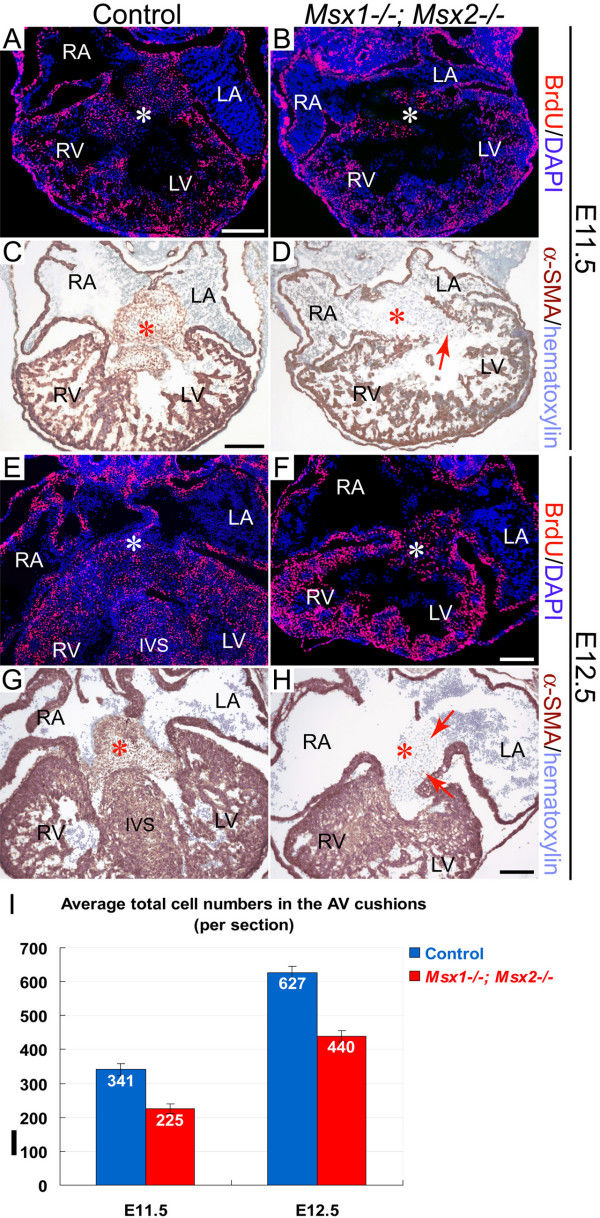
**Normal proliferation but decreased total cell numbers in *Msx1-/-*; *Msx2-/- *mutant AV cushions during EMT**. Immunostaining with the anti-BrdU antibody at both E11.5 and E12.5 revealed comparable levels of cell proliferation in the AV cushions and atrial and ventricular myocardium of *Msx1-/-*; *Msx2-/- *mutants and their littermate controls (compare A with B and E with F). Immunostaining against α-smooth muscle actin (α-SMA) showed dramatically reduced numbers of α-SMA-positive mesenchymal cells in the *Msx1/2 *null mutant AV cushions compared with the control (red arrows in D and H indicate sparse α-SMA-positive cells in the double mutant AV cushions). White asterisks in A, B, E and F, and red asterisks in C, D, G and H indicate the location of the AV cushions. I, Statistical analyses revealed significantly reduced total cell numbers in the AV cushions of the *Msx1/2 *double mutants compared with those of their littermate controls (at least 4 sections were counted for each embryo, and 3 pairs of *Msx1/2 *double mutants and their littermate controls were analysed; *P *< 0.01 for both E10.5 and E11.5, Student's *t *test). IVS, interventricular septum; LA and LV, left atrium and ventricle; RA and RV, right atrium and ventricle. Scale bars: 0.1 mm in A (for A, B), C (for C, D), F (for E, F) and H (for G, H).

We also analysed expression of *Has2*, which is the principle synthase of the extracellular matrix component hyaluronan (HA) [1,36], in the AV cushion mesenchyme of the *Msx1-/-; Msx2-/- *mutants. HA has been demonstrated to mediate endocardial cushion expansion by interacting with water and other extracellular matrix components to create a hydrated low-resistance macromolecular environment that promotes loss of cell contact inhibition and increased cell motility, which is a prerequisite of cardiac cushion expansion and subsequent EMT [1,36,50-52]. Our results indicated normal expression of *Has2 *in spite of a greatly decreased number of *Has2*-expressing cells in the *Msx1/2 *double mutant AV cushion mesenchyme (compare the regions pointed by red arrows in Fig. 3E and 3F). Statistical analyses of multiple sections across the AV cushions revealed that, in spite of normal cell proliferation and apoptosis, there was a significant reduction in the total number of mesenchymal cells in *Msx1-/-; Msx2-/- *mutants compared with their littermate controls (Fig. 4 and data not shown). In addition, we found that there were only sparse mesenchymal cells expressing α-smooth muscle actin (α-SMA) in the *Msx1/2 *double mutant AV cushions, whereas the majority of mesenchymal cells in the control AV cushions are α-SMA-positive (compare the regions indicated by red asterisks in Fig. 4C, 4D, 4G and 4H). As α-SMA was previously shown to be a marker of EMT [39], our results further support impaired EMT in the *Msx1/2 *null mutant AV cushions, which may contribute to a decreased total number of cushion mesenchymal cells.

### Loss of function of both Msx1 and Msx2 perturbs myocardial signaling in the AV canal

**Figure 5:**
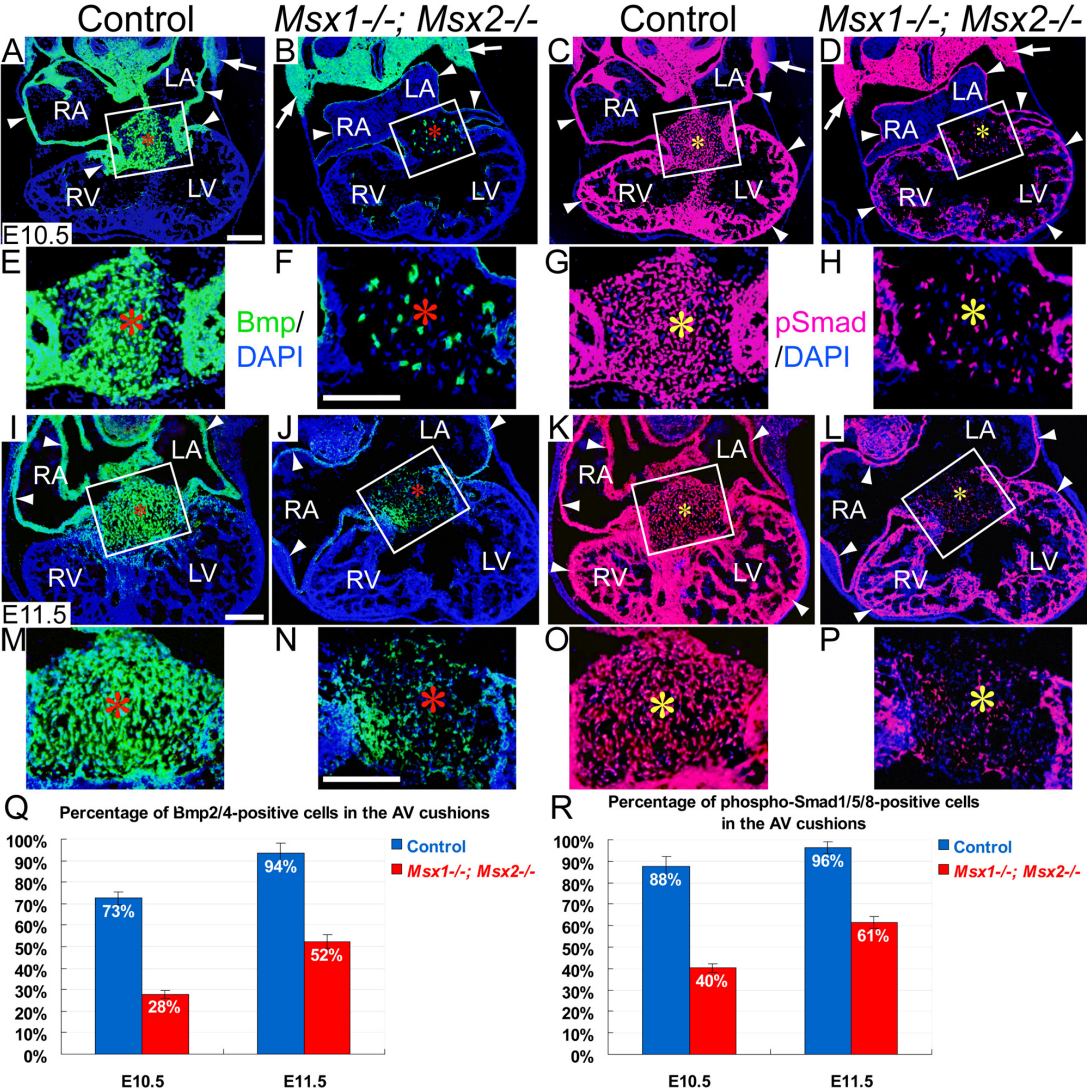
**Reduced Bmp2/4 signaling in *Msx1-/-*; *Msx2-/- *mutant AV cushions and myocardium during EMT**. Panels E-H and M-P are enlarged views of the boxes in panels A-D (at E10.5) and I-L (at E11.5), respectively. Immunostaining for Bmp2/4 (green fluorescence in A, B, E, F, I, J, M and N) and phosphorylated Smad1/5/8 (red fluorescence in C, D, G, H, K, L, O and P) revealed dramatically reduced Bmp2/4-positive and phospho-Smad-positive signals in the *Msx1/2 *mutant AV cushion mesenchyme (red asterisks in B, F, J, N and yellow asterisks in D, H, L, P) and myocardium (white triangle arrowheads in B, D, J and L) compared with the control (A, C, E, G, I, K, M and O). On the other hand, *Msx1-/-*; *Msx2-/- *mutants exhibit broader expression patterns of both Bmp2/4 and Smad1/5/8 in the pharyngeal mesoderm compared with controls (indicated by white arrows in A-D). Q and R indicate average percentages of Bmp2/4-positive and phospho-Smad1/5/8-positive cells in the control and *Msx1/2 *mutant AV cushions at E10.5 and E11.5 (*n *= 3 for each developmental stage, *P *< 0.001, Student's *t *test). LA and LV, left atrium and ventricle; RA and RV, right atrium and ventricle. Scale bars: 0.1 mm in A (for A-D), F (for E-H), I (for I-L) and N (for M-P).

A variety of Bmps (bone morphogenetic proteins), including Bmp2, Bmp4, Bmp5, Bmp6 and Bmp7, have been implicated in regulating EMT during OFT and AV valve formation [16-18,33,40,41,53,54]. As *Msx1 *and *Msx2 *both have been shown to be upstream regulators of *Bmp2*, *Bmp4 *and *Bmp7 *during organogenesis [30,55-57], we asked whether the EMT-regulating Bmp signals were deficient in *Msx1/2 *mutant AV cushions. Immunostaining for Bmp2/4 and phosphorylated Smad1/5/8 at E10.5 and E11.5 revealed significantly decreased Bmp2/4 signaling (more than 50% decrease at E10.5 and more than 30% decrease at E11.5) in the AV cushion mesenchyme of *Msx1-/-; Msx2-/- *mutants compared with their littermate controls (Fig. 5E–H, 5M–P, 5Q and 5R; AV cushions are marked by red and yellow asterisks). In addition to the AV cushions, we also found decreased Bmp2/4 signaling in the atrial and ventricular myocardium of *Msx1/2 *double mutants (indicated by white triangle arrowheads in Fig. 5A–D and 5I–L). Therefore, in contrast to the local upregulation of Bmp2/4 signaling in the *Msx1/2 *null mutant OFT and pharyngeal mesoderm (white arrows in Fig. 5A–D, and our previous study) [32], down-regulation of Bmp2/4 signaling in *Msx1/2 *double mutants is restricted to the AV cushions and chamber myocardium.

**Figure 6:**
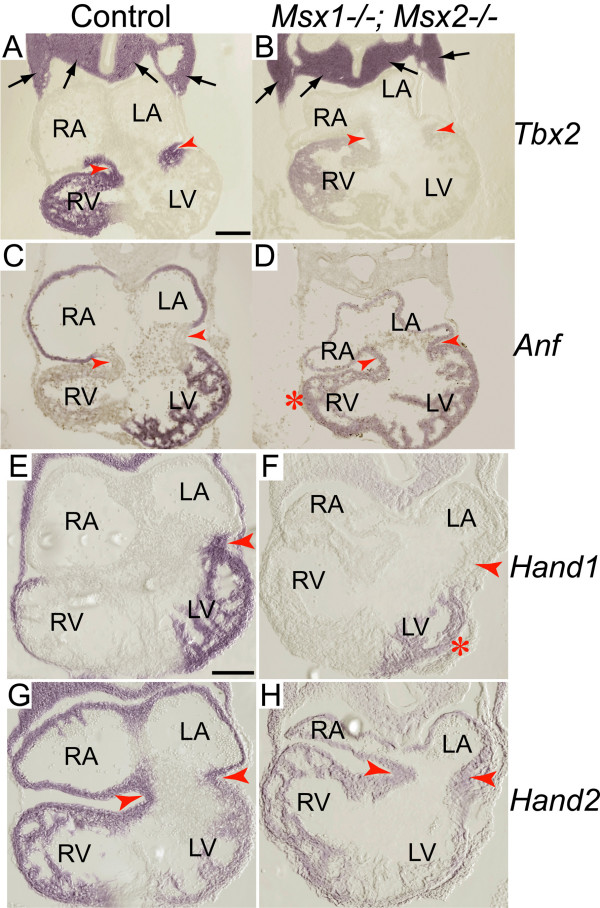
**Perturbed expression of chamber-specific genes in *Msx1-/-*; *Msx2-/- *mutant myocardium**. RNA section in situ hybridization at E10.5 demonstrated that *Tbx2 *expression was dramatically reduced in the *Msx1/2 *null mutant AV myocardium compared with the littermate control (compare the staining intensity in the regions pointed by red notched arrowheads in A and B). In contrast, the level of *Tbx *expression in the pharyngeal mesoderm was comparable between the control and the *Msx1/2 *double mutant (indicated by black arrows in A and B). Concomitant with decreased *Tbx2 *expression in the *Msx1-/-; Msx2-/- *mutant AV myocardium, *Anf *expression exhibited increased expression in the *Msx1/2 *double mutant AV myocardium (compare the staining intensity in the regions pointed by red notched arrowheads in C and D). In addition, we detected ectopic *Anf *expression in the double mutant right ventricle (red asterisk in D), which was likely a hemodynamic effect secondary to the changes of gene expression in the AV canal. *Msx1/2 *null mutants also exhibited decreased expression of *Hand1 *(F) and *Hand2 *(H) in the AV myocardium (red notched arrowheads). In the *Msx1/2 *mutant AV myocardium, *Hand1 *expression was almost undetectable and *Hand2 *expression was significantly reduced compared with the control (red notched arrowheads in E-H). LA and LV, left atrium and ventricle; RA and RV, right atrium and ventricle. Scale bars: 0.1 mm in A (for A-D) and E (for E-H).

Previous studies have demonstrated that *Bmp2 *induces *Tbx2 *(T-box transcription factor 2) expression in the AV myocardium, which in turn inhibits the expression of chamber-specific genes including *Anf *(atrial natriuretic factor), *Chisel *and *Cx40 *in the AVC, and thus establishes the identity of chamber myocardium [17,40,42,43,49]. To investigate whether reduced Bmp2/4 signaling in *Msx1/2 *mutant AVC perturbed gene expression in the AV myocardium, we compared expression of *Tbx2 *and *Anf *in controls and *Msx1/2 *double mutants. In *Msx1-/-; Msx2-/- *mutants, *Tbx2 *expression was dramatically reduced in the AV myocardium (compare the regions pointed by red arrowheads in Fig. 6A and 6B). This is a local down-regulation, as *Tbx2 *expression in the pharyngeal mesoderm was comparable between controls and *Msx1/2 *double mutants (compare the regions pointed by black arrows in Fig. 6A and 6B). In agreement with decreased *Tbx2 *expression, *Msx1/2 *double mutants displayed increased and ectopic expression of *Anf *in the AV myocardium (compare the regions pointed by red arrowheads in Fig. 6C and 6D). *Anf *expression was also increased in the myocardium of the double mutant right ventricle (marked by the red asterisk in Fig. 6D).

**Figure 7:**
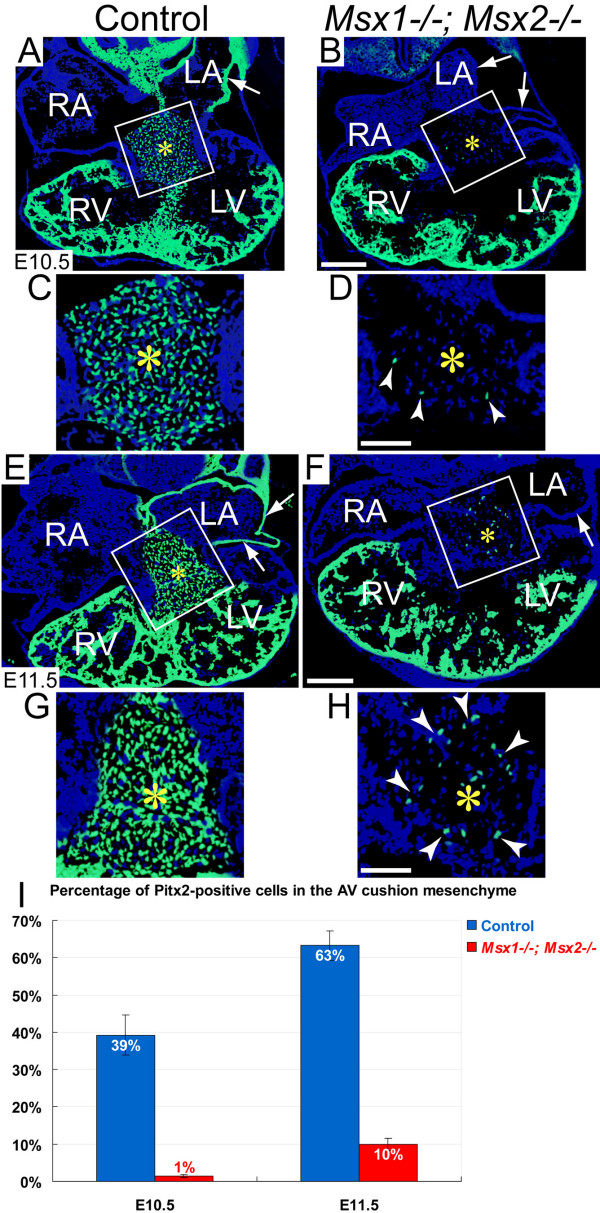
**Decreased contribution of Pitx2-positive cells to the *Msx1-/-*; *Msx2-/- *mutant AV cushion mesenchyme**. Panels C, D, G and H are enlarged views of the boxes in A, B (at E10.5) and E, F (at E11.5), respectively. Pitx2 immunostaining (green fluorescence) revealed a substantial reduction in the number of Pitx2-positive cells in the *Msx1-/-*; *Msx2-/- *mutant AV cushion mesenchyme compared with the control (compare the regions marked by yellow asterisks in C, D, G and H). White notched arrowheads in D and H indicate sparse Pitx2-positive mesenchymal cells in the *Msx1/2 *null mutant AV cushions. There was no detectable Pitx2 expression in the left atrial myocardium of the *Msx1/2 *double mutants shown in B and F (indicated by white arrows). I, Statistical analyses revealed significantly reduced percentages of Pitx2-positive cells in the AV cushion mesenchyme of the *Msx1/2 *double mutants at both E10.5 and E11.5 (*n *= 3 for each developmental stage, *P *< 0.01 at E10.5, *P *< 0.001 at E11.5, Student's *t *test). LA and LV, left atrium and ventricle; RA and RV, right atrium and ventricle. Scale bars: 0.1 mm in B (for A, B) and F (for E, F), 0.05 mm in D (for C, D) and H (for G, H).

In addition to *Tbx2*, other transcription factors that have been implicated in regulation of *Anf *expression, including *Hand1 *[46], *Hand2 *[47], and *Pitx2 *[58], also exhibited dramatically reduced expression in *Msx1-/-; Msx2-/- *mutant AVC (Fig. 6E–H and Fig. 7). *Hand1 *is normally expressed in the left AV myocardium [45,48,59], but this was almost undetectable in *Msx1/2 *double mutants (compare the regions pointed by red arrows in Fig. 6E and 6F). In addition, *Hand1 *expression was significantly decreased in the left ventricular myocardium of the *Msx1/2 *null mutants compared with the controls (marked by a red asterisk in Fig. 6F; three pairs of *Msx1-/-; Msx2-/- *mutants and littermate controls were analysed). Similar to *Hand1*, there was an overall decrease of *Hand2 *expression in the *Msx1/2 *double mutant myocardium (compare Fig. 6G and 6H). In fact, we have previously demonstrated reduced *Hand1 *and *Hand2 *expression in the *Msx1/2 *null mutant OFT and secondary heart field, suggesting that *Hand1 *and *Hand2 *are target genes regulated by *Msx1 *and *Msx2 *[32]. We found that, in contrast to the control AV cushions, where approximately 40–60% of mesenchymal cells expressed *Pitx2 *(Fig. 7C, 7G and 7I; AV cushions are marked by yellow asterisks), there were only sparse *Pitx2*-expressing mesenchymal cells (average ≦10%) in the *Msx1/2 *double mutant AV cushions (Fig. 7D, 7H and 7I). In addition, there was a substantial reduction of Pitx2 immunostaining signal in the myocardium of the *Msx1/2 *double mutant left atrium shown in Fig. 7B and 7F (indicated by white arrows; compare with Fig. 6A and 6E). Multiple lines of evidence have shown that loss of *Hand1 *or *Pitx2 *expression in the AVC cause AV valve defects [37,38,45,60]. Myocardium-specific deficiency of *Hand1 *was shown to cause hyperplastic AV cushions and thickened AV valves [45], implicating *Hand1 *in post EMT valve remodelling [6]. *Pitx2 *may be required for both EMT and post EMT cushion morphogenesis, as *Pitx2 *descendents were reported to be indispensable for late AV cushion formation and AV valve remodelling [38].

## Discussion

Previous studies have demonstrated that *Msx1 *and *Msx2 *are both expressed in the AV endocardium, while *Msx2 *is also expressed in the cushion mesenchyme and AV myocardium [17,33,61,62]. In this study, we demonstrated overlapping expression of *Msx1 *and *Msx2 *in not only the AV endocardium but also the cushion mesenchyme (Fig. 2). In support of the overlapping expression patterns, *Msx1 *and *Msx2 *exhibited redundant functions in the AV endocardium and cushion mesenchyme during EMT. We found that *Msx1 *and *Msx2 *function redundantly to upregulate *NFATc1 *expression in the AV endocardium and maintain *Notch1 *and *Bmp2/4 *expression in the AV cushions during EMT (Fig. 3A–D and Fig. 5). Previous studies demonstrated that NFATc1 signaling during valve morphogenesis is dispensable for EMT (between E8.5 and E10.5) but is required for remodeling of the endocardial cushions into mature valve leaflets (after E11) [9,10,12,63]. NFATc1 was found to regulate valve remodeling by transcriptional activation of matrix degrading enzymes including cathepsin K, whose substrates are collagen and elastin, via the RANKL signaling pathway [63]. Notch signaling in the AV endocardium was reported to promote loss of cell-cell contact via down-regulation of endocardial *VE-cadherin *and induce EMT via upregulation of myocardial *TGF-β2 *[4,22]. To initiate EMT, Bmp2 acts synergistically with TGF-βs to enhance the TGF-β-induced phenotypic changes associated with EMT [16,18,19,40].

We failed to detect any significant change of expression of *Twist1*, which has been implicated in down-regulation of *E-cadherin *and *VE-cadherin *during epithelial- or endothelial-to-mesenchymal transition [17,64,65]. It is likely that the expression levels of other transcription factors which also repress *E-cadherin*/*VE-cadherin *expression, including *Snail *and *Slug *[4,64], are decreased in the *Msx1-/-; Msx2-/- *mutant AV endocardium. In fact, *Snail *expression is activated by Notch signaling [22]. It remains to be determined whether the expression of *Snail *and *VE-cadherin *are perturbed in the *Msx1/2 *double mutant AV endocardium.

In agreement with impaired EMT, we observed significantly decreased total numbers of mesenchymal cells in the *Msx1-/-; Msx2-/- *mutant AV cushions (Fig. 4I). Furthermore, only a small proportion of mesenchymal cells in the *Msx1/2 *double mutant AV cushions expressed α-SMA, suggesting impaired differentiation during EMT (Fig. 4D and 4H). On the other hand, both cell proliferation and cell survival were normal in *Msx1-/-; Msx2-/- *mutant AV cushions during EMT (Fig. 4A, B, E, F and data not shown).

Interestingly, we found decreased Bmp2/4 signaling and expression of *Tbx2*, *Hand1 *and *Hand2 *in the *Msx1-/-; Msx2-/- *mutant myocardium, including the AV myocardium, which expresses *Msx2 *but not *Msx1 *in wild-type embryos, and the chamber myocardium, where neither *Msx1 *nor *Msx2 *is normally expressed. One possible explanation is that perturbed gene expression in the double mutant myocardium is a secondary effect of hemodynamic changes due to the absence of normal AV cushions. Reduced expression of both *Hand1 *and *Hand2 *in the *Msx1/2 *null mutant myocardium further supports our previous hypothesis that *Hand1 *and *Hand2 *are candidate target genes regulated by *Msx1 *and *Msx2 *[32]. Decreased *Hand1 *expression in the *Msx1/2 *double mutant AV myocardium may be associated with defects in remodeling of the AV cushions into mature valve leaflets, since myocardium-specific *Hand1 *deficiency led to thickened AV valves [45], which were previously shown to be associated with impaired valve remodeling [1,6,15].

It is noteworthy that, in contrary to decreased Bmp2/4 signaling in the *Msx1/2 *null mutant AVC and chamber myocardium, our previous study demonstrated increased Bmp2/4 signaling in the double mutant OFT myocardium and cushion mesenchyme [32]. It has been shown that *Bmp2 *expression is normally switched from the OFT myocardium to the AVC and atrial myocardium between E9.5 and E10.5 [16,61,66], suggesting that an alternative explanation for perturbed Bmp signaling in *Msx1-/-; Msx2-/- *mutant hearts is that *Bmp2 *expression does not shift from the OFT to the AVC in the double mutants. Locally reduced *Bmp2 *expression in the *Msx1/2 *null mutant AVC may be insufficient to maintain normal expression of *Tbx2 *and in turn lead to reduced *Tbx2 *and increased *Anf *expression in the double mutant AV myocardium (Fig. 6A–D) [17,40,42,43,49]. Perturbed *Bmp2 *expression may also disrupt normal *Pitx2 *expression in the *Msx1-/-; Msx2-/- *mutant AVC and atrial myocardium, as *Bmp2 *has been shown to be a positive regulator of *Nodal *signaling and *Pitx2 *[67]. In addition, there may be altered expression patterns of other Bmp molecules, including *Bmp6*, that contribute to the abnormal distribution of Bmp signals in the *Msx1-/-; Msx2-/- *mutant OFT and AV cushions. Interestingly, *Bmp6 *exhibits an asymmetric (left-sided) expression in the OFT myocardium at E10.5, reminiscent of the expression pattern of *Pitx2 *[53]. *Bmp6 *expression undergoes a transition from the AV cushion mesenchyme to the OFT cushion mesenchyme between E10.5 and E12.5, indicating that it plays a critical role in both the EMT of the AV cushions and the development of the OFT cushions [16,53]. Further analyses of the expression patterns of the aforementioned Bmp ligands will determine which Bmp molecules are critical contributors to the impaired development of the *Msx1-/-; Msx2-/- *mutant OFT and AV cushions.

## Conclusion

In this study, we documented redundant functions of *Msx1 *and *Msx2 *genes in distinct aspects during AV cushion morphogenesis: endocardial activation prior to EMT (via upregulation of *NFATc1 *expression), induction of EMT (via upregulation of Notch1 and Bmp2/4 signaling in the AVC), as well as post-EMT valve remodelling (via upregulation of *Hand1 *expression in the AV myocardium).

## Methods

### Mouse strains and genotyping

All mice used in this study were maintained in a mixed genetic background of BALB/c and CD-1. *Msx1-/- *and *Msx2-/- *single knockout and *Msx1-/-; Msx2-/- *double knockout mice were described previously [30,34,35]. The noon copulation plug was counted as embryonic day 0.5 (E0.5). Genomic DNA was extracted from yolk sac (embryos) or tails (postnatal mice) for genotyping. PCR primers and conditions for *Msx1 *and *Msx2 *knockout alleles as well as the *Wnt1-Cre *and *R26R *transgenes were as described [34,35,68].

### Histology, section in situ hybridization and immunostaining

For histology, embryos at E15.5 were fixed with 4% paraformaldehyde (PFA) in phosphate-buffered saline (PBS) for 14 hours at 4°C, dehydrated through graded ethanol and embedded in paraffin wax. Sections were cut at 7–8 μm and stained with Hematoxylin and Eosin. For cryostat sectioning, fixed embryos at E10.5 were dehydrated through graded methanol and stored in 100% methanol at -20°C till use. Stored embryos were rehydrated through graded methanol, washed in PBS, and cryopreserved in sucrose solutions with increasing concentrations, then frozen in O.C.T Sections were cut at 10-μm thickness and RNA section in situ hybridization was performed using the In Situ Hybridization kit (BioChain) with the protocol modified according to Dijkman et al. [69]. All RNA probes were generated as described: *Anf *[17], *Hand1 *[45], *Hand2 *[45], *Has2 *[17], *Notch1 *[17], *Pitx2 *[38], and *Tbx2 *[17].

For immunostaining, the following primary antibodies were used: biotinylated goat polyclonal anti-BMP-2/4 (1:100, R&D Systems), mouse monoclonal anti-bromodeoxyuridine (BrdU) (1:100, Sigma), mouse monoclonal anti-NFATc1 (1:200, BD Pharmingen; a kind gift from Dr. Kaartinen), guinea pig polyclonal anti-Pitx2 (1:200, a kind gift from Dr. Kioussi [70]), rabbit polyclonal anti-phospho-Smad1/5/8 (1:50, Cell Signaling).

Littermates of each *Msx1-/-; Msx2-/- *mutant embryo were used as the controls, and no discernable variation in either gene expression or cell proliferation/survival/differentiation was detected between the controls from the same litter and with different genotypes.

## Abbreviations

Anf: Atrial natriuretic factor; AV: Atrioventricular; Bmp: Bone morphogenetic protein; EMT: Endothelial-mesenchymal transformation; NFAT: Nuclear factor in activated T cells; PBS: Phosphate-buffered saline; PFA: Paraformaldehyde; TGF: Transforming growth factor.

## Authors' contributions

Y–HC designed most of the experiments, carried out mouse dissections at E10.5 and E11.5, analyzed the results and drafted the manuscript. MI performed mouse dissections and histological analyses at E15.5, and first observed the phenotype of malformed AV valves in *Msx1-/-; Msx2-/- *double mutants. HMS diagnosed the AV cushion and valve defects in *Msx1/2 *double mutant mice. REM conceived, funded and supervised the project, which was carried out in his laboratory. Both HMS and REM critically read and revised the manuscript. All authors have read and approved the final manuscript.
